# Predicting species emergence in simulated complex pre-biotic networks

**DOI:** 10.1371/journal.pone.0192871

**Published:** 2018-02-15

**Authors:** Omer Markovitch, Natalio Krasnogor

**Affiliations:** Interdisciplinary Computing and Complex Bio-Systems research group, School of Computing Science, Newcastle University, Newcastle upon Tyne, United-Kingdom; Santa Fe Institute, SPAIN

## Abstract

An intriguing question in evolution is what would happen if one could “replay” life’s tape. Here, we explore the following hypothesis: when replaying the tape, the details (“decorations”) of the outcomes would vary but certain “invariants” might emerge across different life-tapes sharing similar initial conditions. We use large-scale simulations of an *in silico* model of pre-biotic evolution called GARD (Graded Autocatalysis Replication Domain) to test this hypothesis. GARD models the temporal evolution of molecular assemblies, governed by a rates matrix (i.e. network) that biases different molecules’ likelihood of joining or leaving a dynamically growing and splitting assembly. Previous studies have shown the emergence of so called compotypes, i.e., species capable of replication and selection response. Here, we apply networks’ science to ascertain the degree to which invariants emerge across different life-tapes under GARD dynamics and whether one can predict these invariant from the chemistry specification alone (i.e. GARD’s rates network representing initial conditions). We analysed the (complex) rates’ network communities and asked whether communities are related (and how) to the emerging species under GARD’s dynamic, and found that the communities correspond to the species emerging from the simulations. Importantly, we show how to use the set of communities detected to predict species emergence without performing any simulations. The analysis developed here may impact complex systems simulations in general.

## Introduction

The Origins of Life (OOL) field attempts to understand the transition from a mixture of life-less molecules to life-full entities, with protocells [1–4] as intermediate (potentially viable) milestones along the non-living to living spectrum [5]. A widely accepted definition of minimal life is: a self-sustaining system capable of undergoing Darwinian evolution [6], while other definitions are often similar (e.g. [7]). A minimally living entity needs not be a cell as we know it but could be a much simpler protocell [2, 8–15], i.e. container with some necessary molecular content. Two major schools tackle the problem of transition from non-life to life: the genetic, or replicator-first approach, and the metabolism-first approach. The replicator-first approach focuses on a single self-perpetuating informational biopolymer, e.g., RNA, as the first step, and it is thus often referred to as the “RNA world” [16–20]. In contrast, the metabolism-first approach [2, 9, 11, 21–23] focuses on a network of chemical reactions among simpler chemical components that became endowed with some reproductive characteristics [2, 8, 9, 11–13].

The RNA world, a widely accepted replicator-first scenario, assumes that a molecule similar or analogous to present day RNA played the role of the self-perpetuating biopolymer [17–19, 24]. The mixture of such molecules is assumed to have later evolved both a metabolic network and an encompassing container. The RNA-world draws from RNA’s capability to store (sequential) information and certain catalytic activities typical of metabolism [25–28].

The metabolism-first scenario, on the other hand, suggests that the very first life precursors are likely to have been relatively elaborate molecular networks of much simpler organic molecules, thus trading the complexity of the building blocks (e.g. RNA) for the complexity at the ensemble level. One of the first suggested possible chemical pathway for the emergence of life was made by Oparin, who proposed that it could be manifested by the molecular reactions of relatively simple organic molecules in the primordial soup, interacting with each other to spontaneously form colloidal molecular assemblies (coacervates) [8, 29, 30].

The lipid world scenario for OOL is a variant of the metabolism first scenario, which considers a complex chemical system consisting of mixture of mutually interacting simple molecule types which spontaneously form noncovalent assemblies [22, 31]. Importantly, these assemblies store information in the form of non-random molecular compositions–compositional information (i.e. the specific ratio of different molecule types that make up the assembly)–and pass it to progeny via homeostatic growth accompanied by fission. This information transmission is a function similar to what can be done with sequence-based biopolymers such as RNA/DNA/PNA, except that in this case it is compositional information that is preserved and propagated rather than sequential information. Specifically, compositional replication is the transfer (at least partially) of compositional information from parent to progeny, where the process of information transfer is itself a function of the compositional information in the parent entity [32]. The composition encoded in several chemical systems has been shown to affect their physical properties (i.e. phenotypes), supporting the realism of the lipid world. For example, vesicles’ lipid-composition has been shown to affect dye encapsulation efficiency [33] or vesicle’s structure [34], and genetic programming (“evolutionary algorithms”) has been applied to evolve vesicles’ formulation [35, 36]. More recently it has been suggested that vesicles can “osmotically” couple otherwise decoupled chemical reactions [37].

**Fig 1:**
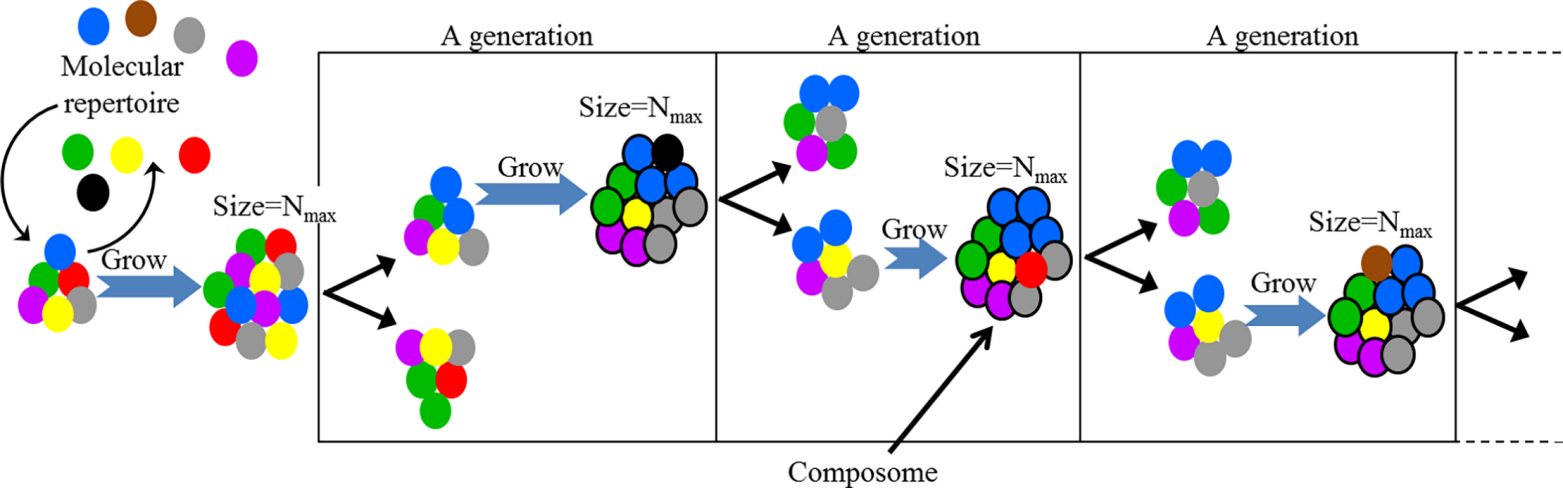
Schematics of GARD’s dynamics. Different molecules types (represented by different colored circles) aggregate to form assemblies. Aggregation is biased by a matrix of chemical rates (β, Eq 1)). Once an assembly reaches a size-threshold (N_max_) it splits, and the progeny then continues the growth-split cycles (generations). A composome is an assembly that has high average compositional similarity (see section: The GARD model) to its parent and to one of its children.

The GARD kinetic model is a physio-chemical simulator within the lipid world scenario [31, 38–40]. The model is based on a matrix (named β) that determines the interactions between different molecular types while the system is kept away from thermodynamic equilibrium by assembly fission (Fig 1). GARD dynamics exhibit quasi-stationary states, which appear in the simulation as faithfully replicating molecular assemblies, termed composomes (for compositional genomes) [38]. Clusters of compositionally-similar composomes are called compotypes (for composome types) [41]. These compotypes have been shown to respond to selection [40], exhibit ecology-like population dynamics [42] and exhibit quasispecies behavior including error-catastrophe-like transition [32] and hence have been interpreted as (emergent) species.

The next paragraph presents a more elaborate discussion of selection in GARD, which can be summarised as the following: GARD simulations show compotypes (but note that not every composition is a compotype), these compotypes can respond to external selection (but not always) by changing their frequencies within a population. Under very small alphabet size and very small assembly size this change in frequency seems negligible.

As typical GARD simulations take a constant number of alphabet molecule types and a predefined assembly size, the total number of possible compositions is fixed [32] and the system is not permitted to show true open-ended evolution [43]. In 2010, perhaps the first rigor attempt at studying evolution in GARD was reported, in the sense of population responding to an external selection pressure [44]. Unfortunately, the study was based on a single instantiation of a random lognormal matrix, which hinders on the ability to draw conclusions from it. Moreover, the study employed parameters values very different than those typical used in GARD (i.e. small alphabet size and small assembly size), and the study did not designate compotype species as targets for selection. A later study considered a similar methodology for selection as the 2010 paper, and explored a large number of matrix instances and focused on compotypes as selection targets **[40]**, asking whether compotypes change their frequency within a population as an outcome of external selection. The later study found that GARD systems can respond to selection (but not always), and that this selection response is more favourable when the matrix instance is highly mutualistic (i.e. when off-diagonal values are higher than diagonal values). A recent attempt to extend the 2010 paper by attempting to map GARD into the quasispecies formalism [45] presents an argument on GARD’s putative limited evolvability. The paper failed however to designate compotypes as selection targets, even thought it was previously shown that only compotypes can be mapped into quasispecies [32], and used atypical GARD parameters.

Regardless of selection behaviour, the present paper asks whether the biological diversity that surrounds us would be different if the tape of life was to run again from the start [46–49] under similar initial conditions, and whether adaptations that lead to similar phenotypes follow a quantifiably repeatable route [50]. Some evidence for the convergent nature of evolution can be seen when two separated populations of *E*. *coli* evolved separately for many generations in identical environments achieved similar fitness [51], or when different populations of lizards from different nearby islands developed into similar ecomorphs independently [52]. Computer models have also been used to study this question [53–58].

**Fig 2:**
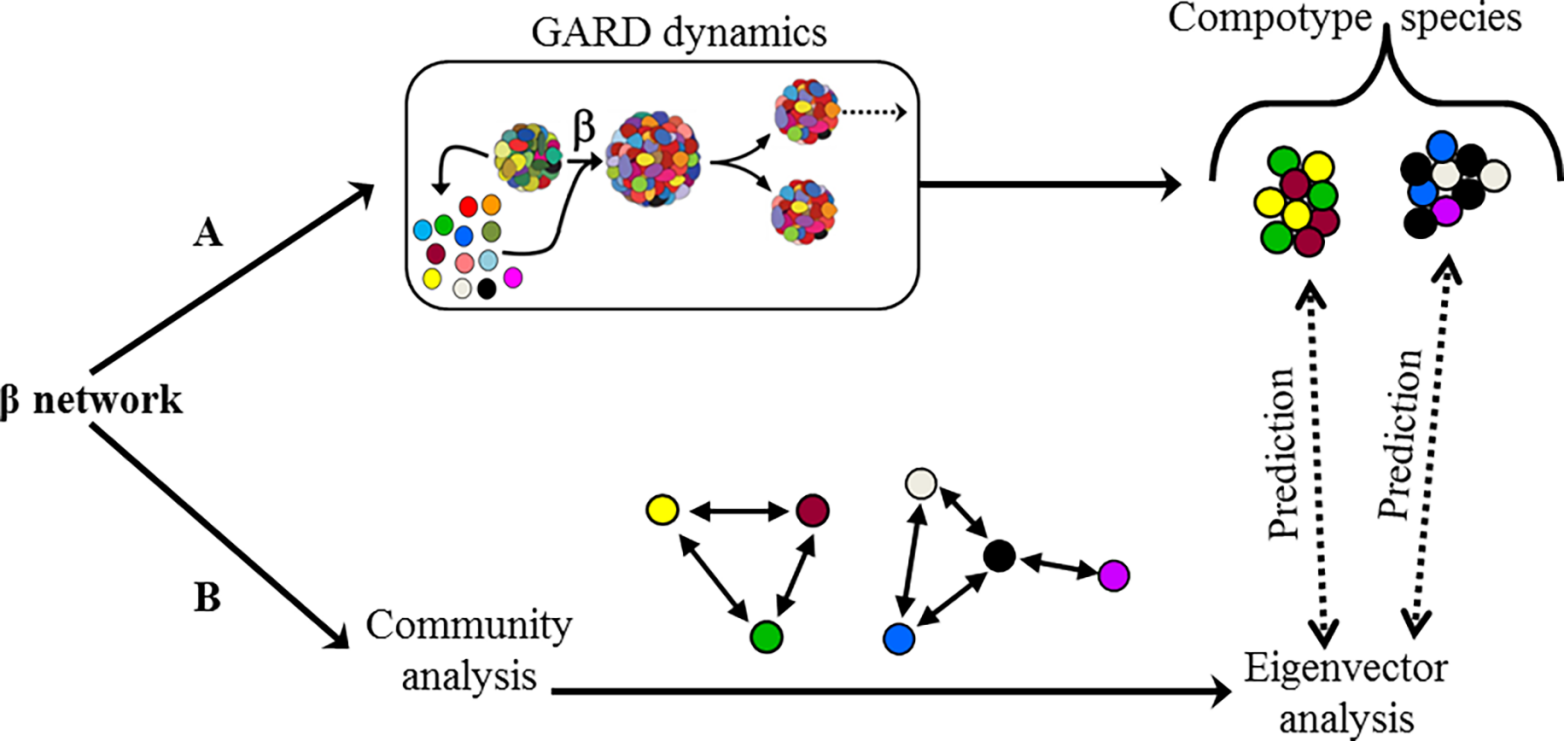
Overview of the algorithm developed in the present work. (**A**) A network (β) is employed in GARD simulations and the emerging compotype species are collected. (**B**) In parallel, the communities of β are analysed and collected. Finally, (A) and (B) are compared by using the ensemble of detected communities to predict compotypes.

In this paper we postulate that if evolutionary diversity is dominated by “invariants” rather than “decorations” then it should be possible to predict the outcome of the evolutionary process without actually waiting for it to happen. That is, it should be possible to predict which species will emerge. In the present paper, this translated to investigating the degree to which the emergence of GARD species, i.e. compotypes, can be analysed in terms of β’s inner organization only (i.e. independent of the dynamics in GARD) (Fig 2). In order to do this, we analysed the community structure of β. Typically in a network representation, nodes symbolize entities (molecules, web pages, people, etc`) and edges are relations between the entities (catalysis, hyperlinks, friendship, etc`). Communities are organizational features in many networks, and are generally defined as sets of nodes more densely interconnected between themselves than to other nodes in the network [59–61]. Communities detection algorithms allow revealing of essential internal network organization and typically detection algorithms try to optimise the ratio between the number of internal community to cross-communities edges across all communities simultaneously.

Network science is often fruitfully applied to decipher and understand complex systems, including food-webs [62], metabolic networks [63], genes networks [64], protein networks [65] and different social networks [66, 67]. Such applications of network science, together with previous linear algebra analysis of β [39] and of other networks [68, 69], motivate us to apply such analyses to our system, focusing on how the inner organization of a β affects the nature of observed compotypes species. Even though differences exist between replicating polymers and replicating catalytic networks [32], in both cases the model can be represented as a network [13] and encourages understanding how network’s inner organisation affects the nature of observed species. We showed in [70] that one can predict the best simulation algorithms for systems and synthetic biology models by analysing their network structure. Further, different β’s result in different GARD simulations giving rise to different compotype species provides additional motivation for our current study.

In this paper we use large scale simulations and data analysis of GARD simulations to demonstrate that communities’ analysis allows us to “shortcut” expensive dynamical simulations of a (proto) evolutionary process and predict its invariants, namely, the set of species that can be expected to emerge from such a dynamical system.

## Methods

### The GARD model

GARD describes the growth and fission of a molecular assembly, typically assumed to consist of a large repertoire of amphiphilic molecules drawn from a repertoire of N_G_ molecular types [38, 40] (Fig 1). Molecules from the environment join an assembly and molecules within the assembly it can leave. Once the number of molecules in an assembly reaches a pre-defined size threshold (N_max_), a random fission event takes place and produces two daughter assemblies of the same size (N_max_/2) which can then repeat the growth-fission cycle (Fig 2 show a scheme of the model, adapted from [32]). This dynamic is described by a set of ordinary differential equations:

$$\frac{dn_{i}}{dt}=\left(k_{f}\rho_{i}N-k_{b}n_{i}\right)\left(1+\sum_{j=1}^{N_{G}}\beta_{ij}\frac{n_{j}}{N}\right)$$ Eq 1

Where n_i_ is the current count of molecule type i in an assembly (i = 1..N_G_), k_f_ and k_b_ are the basal forward and backward rate constants (assembly joining and leaving, respectively). ρ_i_ is the buffered environmental concentration and N is current assembly size (N = ∑n_i_). β_ij_ is the rate-enhancement exerted by an assembly molecule of type j on incoming or outgoing molecule of type i.

β can be represented as an N_G_×N_G_ adjacency matrix for a weighted-directed-asymmetric-network with N_G_ nodes and N_G_^2^ edges. Typically, β_ij_ values are drawn from a lognormal distribution [39, 71] (that is, the values ln(β_ij_) are normally distributed with mean = -4 and standard deviation = 4) where different β instances represent different potential environmental prebiotic chemistries [40]. Introducing negative β_ij_ values, i.e. inhibition, is expected to result in catalysis aswell via inhibition of inhibitor [40].

As mentioned previously, composomes are faithfully replicating assemblies, that is a composome is an assembly with high similarity to its predecessor and successor (typically compared when both assemblies are at size N_max_). It is important to distinguish composome assemblies from non composomes (i.e. drifting assemblies), because the latter may appear spontaneously yet are incapable of transmitting compositional information (i.e. the specific ratio of different molecule types): that is, once a non composome assembly reaches the critical size triggering the fission event (N_max_), its compositional information is not preserved in the daughter assemblies and hence is lost. Composomes are grouped into compotypes using k-means clustering algorithm based on compositional similarity as a distance measure (see section: Compotype-community assignment) by picking the k which give the highest silhouette [41]. A compotype is thus represented by a vector constituting the center of mass of all its member assemblies and is interpreted as a GARD species.

### GARD simulations

The GARD model was run using a stochastic kinetic Monte Carlo simulation based on Gillespieʼs algorithm [72] using parameter values identical to those employed in previous studies [32, 40, 42]: k_f_ = 10^−2^, k_b_ = 10^−4^,ρ_i_ = 10^−2^, N_max_ = 10^2^ and N_G_ = 10^2^, for 5,000 growth-split cycles (generations). Calculations were executed using MATLAB version R2015a. A large set of 10,000 GARD simulations was generated, all with the above parameters, and each with a different β, created by MATLAB’s pseudorandom number generator with seeds 1–10,000. Each of these β‘s represents different chemistries that might lead to the emergence of one or more compotypes.

In the basic form employed for this paper, GARD was run in a single-lineage mode, where at each split event only one progeny (picked at random) is followed and the other one is discarded (Fig 1). For each simulation under a given β, composomes where identified and clustered into compotypes.

Simulations give rise to the emergence of various compotype species as a result of the different chemistries represented by different β‘s. The number of compotypes observed in each simulation typically ranged from 1–6, with a total of 20,235 compotypes observed in 10,000 simulations performed (3 simulations out of those failed and were therefore discarded).

We provide the MATLAB code and datasets used in this work (see S1 File (Supporting Information) and reference [73]).

### Community detection algorithms

A community detection algorithm was run on each β, and the list of nodes (molecule types) belonging to each community was recorded per each β. The three different algorithms used are: Louvain (MATLAB version) [74], Infomap (version 0.18.2) [75] and OSLOM (Order Statistics Local Optimization Method) (version 2.5) [76], with their default parameters.

Louvain is a heuristic method to find communities [74]. This method starts by assigning each node to its own community. Then, a node m is added to the community of node n only if this results in increased modularity value. m and n pairs are picked to give the highest increase. This is continued until no increase in modularity is gained by joining nodes. Next, a new network is created, whose nodes correspond to the previously found communities and whose edges are the respective sum of the previous edges between communities. This entire process is repeated until no further increase in modularity is possible.

Infomap is based on flow and encoding [75]. This method first simulates a random walk along the network, biased by the edges' weights. These random walks are then encoded into binary string in a way that would reflect how frequency adjacent nodes are visited, rather than create a maximally compressible binary string. This is done in a two-level description whereby a community of nodes where the walker has spent long periods of time receives unique code, but the nodes within a community receive non-unique codes that can be repeated in other communities’ nodes. In other words, the random walk is efficiently encoded in a way in which important structures (communities) indeed retain unique codes.

OSLOM is finding clusters which are statistically significant with respect to a random network with similar characteristics as the actual network [76]. This method begins by randomly picking a node as the first community and additional nodes are added to this community if they are considered significance in the statistical sense. This is then repeated with other nodes until all communities are found.

## Results and discussion

### GARD tapes

**Fig 3:**
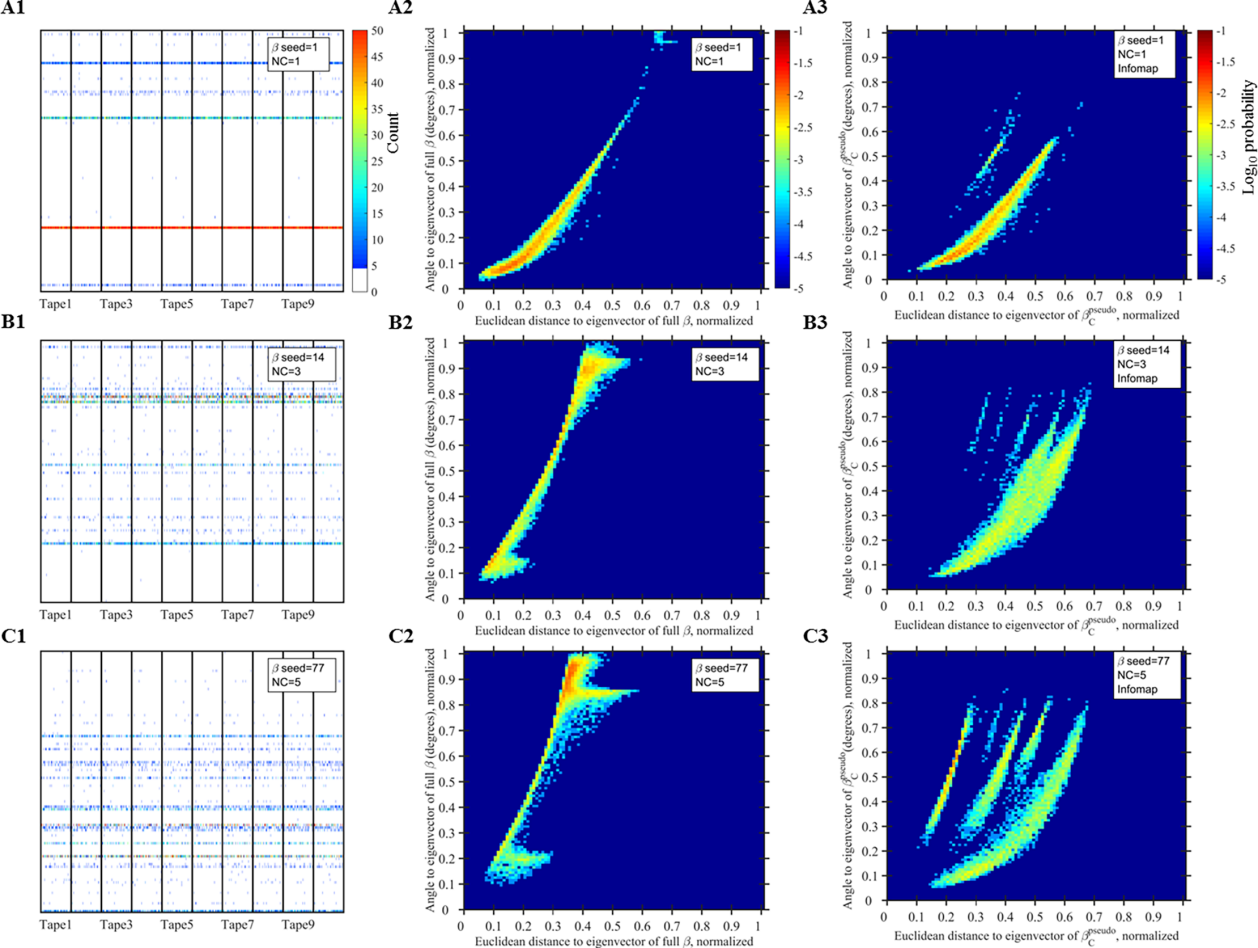
Examples of GARD simulations under different β’s. (A1-C1) Histories of different tapes; For each tape, assemblies from different generations are plotted along the X axis, and color represents the counts of each of the N_G_ molecule types in each assembly (recorded at assembly size N_max_ (Fig 1). Tapes are separated by a vertical black line. For each tape, the first 1,000 assemblies are shown. Red color represents counts ≥ 50, and for brevity counts < 5 are colored white. (A2-C2) Density plots; For each assembly shown in panels (A1-C1), its Euclidean distance and angle vs. the eigenvector of the full-β was calculated (normalized for the maximum value between two assemblies, $N_{\max}\sqrt{2}$ for distance and 90 degrees for angle). Color is normalized probability (log_10_ scale) of an assembly having a certain angle and distance. See section: Compotype-community assignment. (A3-C3) Same as (A2-C2), except for each assembly the distance and angle are calculated against the one eigenvector of β* which has the lowest angle to this assembly. Number of Infomap communities detected is: 9 (A3), 7 (B3) and 6 (C3). Further examples are available at http://ico2s.org/data/extras/gard/ and [73].

In order to understand if convergent evolution is occurring under GARD dynamics, simulation-runs were repeated 10 times under a given β, with different random seeds (and hence initial assembly) each time. Each repeated run is regarded as a GARD “tape” (analogue to replaying the tape of life under the same chemistry (i.e. β)). The history of each tape was recorded (i.e. the content of each assembly) and compotypes were identified for each tape (i.e. k-means clustering). Fig 3 (panels A1-C1) show individual examples of GARD tapes (more examples are available at http://ico2s.org/data/extras/gard/). These panels show the content of assemblies from the different tapes, where different assemblies are plotted along the X axis and the N_G_ molecule types are of each assembly are given along the Y axis, with color representing the count of a molecule type in an assembly. While the detailed histories of various tapes under a given β are different, they generally show similar trends (invariants) represented by the horizontal lines. Further, different tapes from the same β exhibit the same number of compotypes (in 85% of cases studied for this part, see Fig A in S1 File), and, importantly, those compotypes are extremely similar between different tapes (Fig B in S1 File), signifying that GARD dynamics display convergent evolution. In other words, even if different GARD tapes portray different histories (decorations) under the same β, they give rise to very similar compotype species (invariants) and thus it becomes relevant to ascertain to what degree it is possible to predict the emergence of these species from the underlying chemistry alone, i.e., ignoring the dynamical process that generates the species. Because GARD exhibits convergent evolution, in the next sections only a single tape will be simulated per each β, but in return a large number of different β’s will be employed.

### Communities detection

This section presents how community detection algorithms were applied to the β network, and the next section presents how the detected communities were related to the emergence of species in the prebiotic evolution model (GARD) (Fig 2). In order to adequately compare a detected community to an observed compotype one needs to convert a community–which is a set of molecule types (nodes and their links)–to a composition, that is–the ratios between those molecule types. This composition can then be directly compared with the composition of a compotype. To detect communities within different β matrices, each of the three different algorithms used (Louvain [74], Infomap [75] and OSLOM [76]), were run on each β, and the list of nodes (molecule types) belonging to each community was recorded per each β.

**Fig 4:**
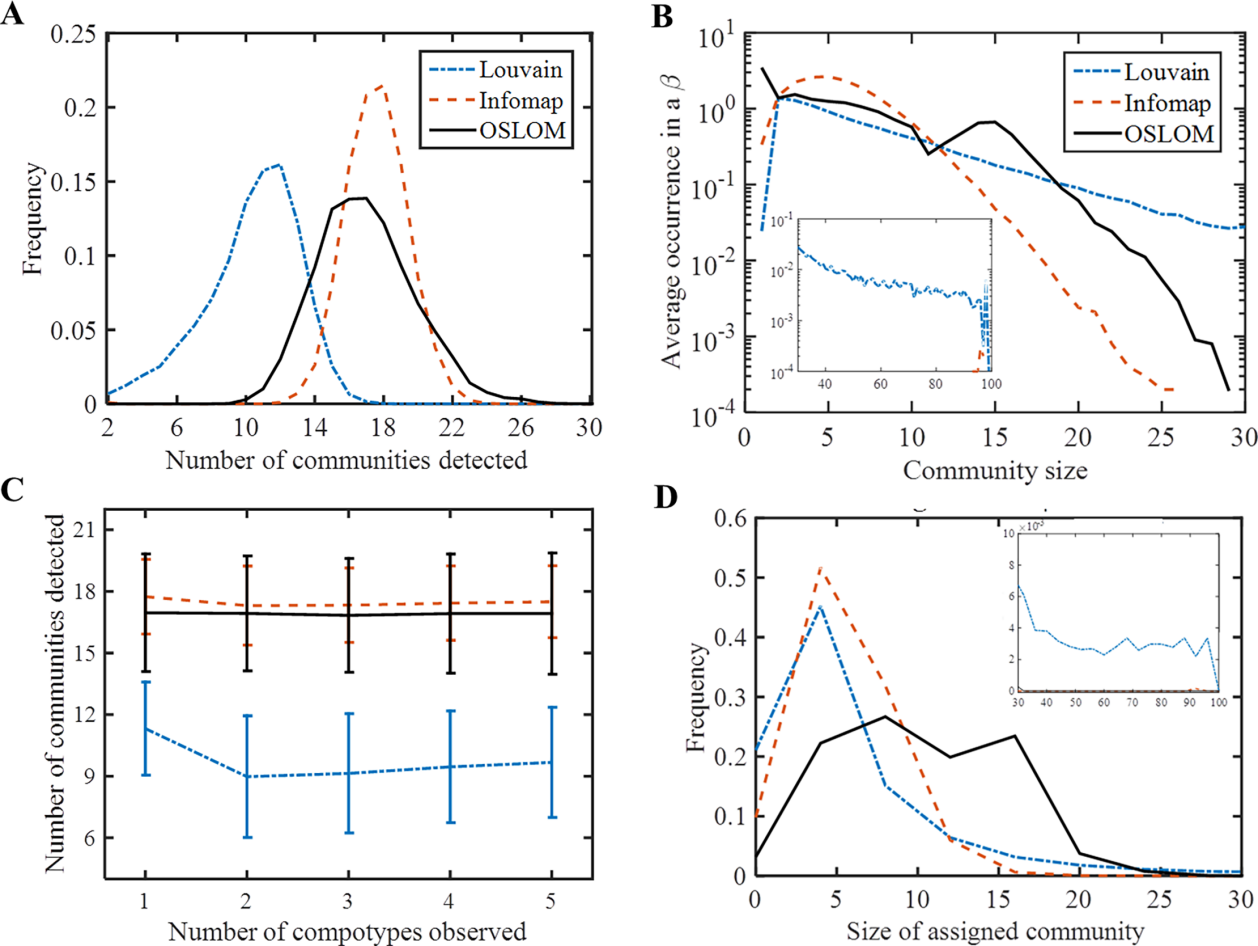
Communities in β’s. (A) Histogram of total number of communities detected in each network, for the 3 algorithms. Frequency is given out of the 10^4^ β’s studied here. (B) Average occurrence of community-sizes. An occurrence of 10^−4^ means that this community-size appeared only in 1 β out of the 10,000 studied here and an occurrence of 1 means that on average each β has one community with this size. Insert show the occurrence of sizes > 30. (C) Average number of communities detected vs. number of observed compotype species shows no correlation. Vertical bars mark standard deviation. (D) Histogram of the size of assigned communities, when a community is assigned to a compotype based on eigenvector similarity (see section: Compotype-community assignment). Mean and standard deviation are given in Table 1.

Each of the three algorithms always detected several communities (>1) in each of the 10,000 different β‘s studied here (Fig 4 A and 4B). Louvain algorithm detected on average fewer communities than Infomap or OSLOM. Interestingly, both OSLOM and Infomap detected similar numbers of communities, even though OSLOM allows for overlaps (i.e. molecules belonging to more than 1 community). The latter suggests that a detection algorithm may sometime consider two overlapping communities as one, if overlaps are allowed. In GARD these overlaps are suggested to be the facilitators of species interconverting into each other–a phenomenon best seen in GARD populations [42].

Different simulations under different β’s give rise to different compotypes, which calls for the search for a link between the inner structures of a β to the emerging compotypes in a simulation under this β. However, as the average number of communities detected in a β is higher than the average number of compotypes observed in a simulation under this β and no correlation between number of communities to number of compotype exists (Fig 4C), finding such link is not trivial. The next section will discuss a methodology for community–to–compotype assignment and prediction.

### Compotype-community assignment

In order to perform such comparison, compotypes observed in each β-dependent simulation were collected (will sometime be referred to as original compotypes) and on the other hand the communities detected in each β were collected (respectively corresponding to (A) and (B) in Fig 2). Then, for each detected community in each β, a matrix β* is created with elements β_ij_*:

$$\beta_{ij}^{*}=\{\begin{array}{cc} \beta_{ij} & i\in C\;\mathrm{AND}\;j\in C\\ 0 & otherwise \end{array}$$ Eq 2

Where C is the set of the indices of all nodes (molecule types) that belong to a community, i and j are nodes’ indices and β_ij_ are elements of β (Eq 1). β* has the same dimensions as β. That is– β* is a sparser version of β matrix in which only pairs of molecule types that belong to a community can interact (all other rates are set to zero). This particular formulation of β* was picked such that its eigenvectors will have the same dimensionality as the original compotypes. Next, linear algebra is used on β*.

According to the Perron-Frobenius theorem a matrix such as β* or β has a nondegenerate largest real eigenvalue with a corresponding eigenvector with all non-negative elements [77, 78]. Indeed, an eigenvector analysis on all the β*’s and β’s studied here showed that only a single non-negative eigenvector exists for each. It is tempting to consider an eigenvector with all non-negative elements as representing a molecular composition (as sometimes done [39, 77, 79]), homologue to a compotype. A vector with some negative elements, representing negative molecular counts or concentrations, by definition cannot represent molecular composition. Because GARD simulations can exhibit more than 1 compotype (Fig 4C), it is unclear what is the relation between the single eigenvector of β to the observed compotypes and the same can be said about the communities. What follows presents a method to successfully predict the content (i.e. composition) of all compotypes observed in a simulation under a given β, given only the ensemble of communities of that β.

The Perron-Frobenius theorem was applied to all β* and the eigenvectors were recorded. Exploring the role of communities in the actual GARD dynamics, the angle and distance between each assembly during a simulation to the eigenvector of the full-β and to the eigenvector of β* were calculated (Fig 3 panels A2-C2 and A3-C3, respectively). Indeed, the assemblies show a lower angle to β* than to β (see also Fig C in S1 File), symbolizing the significance of communities in analysing GARD’s dynamics.

Each such eigenvector of β* is compared with each compotype, using cosine of compotype vectors as typically applied in GARD studies [38, 41, 44, 80]:

$$H\left(V_{1},V_{2}\right)=\cos\left(V_{1},V_{2}\right)=\frac{V_{1}\cdot V_{2}}{\left|V_{1}\right|\cdot \left|V_{2}\right|}$$ Eq 3

H measures how well an eigenvector matches a compotype’s content (i.e. composition), where a value of 1.0 means identical compositions (i.e. one vector is the other vector multiplied by a positive scalar). Each compotype is then assigned with the community that give rise to the highest H.

**Fig 5:**
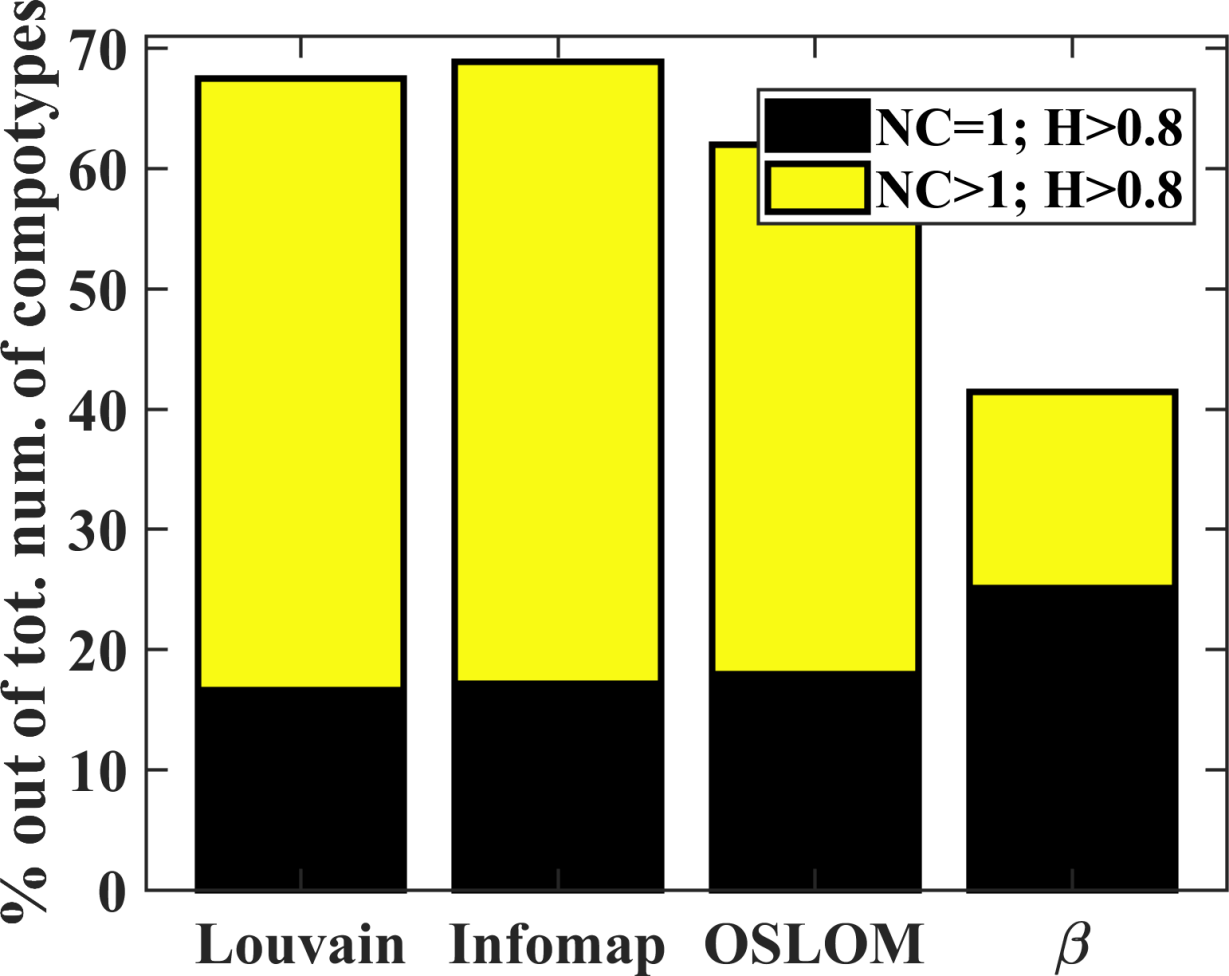
Bar plot of the percentage of high compositional similarity (H, Eq 3) when predicting compotypes using the eigenvectors of β* (Eq 2) vs. full-β. Percentage is given out of the total number of compotypes observed under all β networks. Mean and standard deviation are given in Table 1 and full histograms are given in Fig D in S1 File.

Fig 5 shows, out of all the H values between the communities’ eigenvectors and the original compotypes, the percentage of particularly high values (H>0.8). Full histograms are given in Fig D in S1 File. When multiple compotypes were observed in a simulation, the eigenvectors of β* showed a high degree of similarity to all compotypes whereas the eigenvector of the full-β showed much lower similarity values (Table 1). Only in the limiting case, when only a single compotype is produced by the simulation, the eigenvector of the full-β showed high similarity to that compotype. Two-sample Kolmogorov-Smirnov tests were performed, with the null hypothesis that the similarities with respect to the full-β are from the same continuous distribution as the similarities with respect to β*, against the alternative hypothesis that they are from different continuous distributions. The Kolmogorov-Smirnov tests were repeated for the cases of single and multiple compotypes, for each of the three community detection algorithms (that is– 6 tests in total). All the tests rejected the null hypothesis with alpha level that is essentially zero. Further, when taking into account the overall dataset (that is, without distinguishing between cases with single or multiple compotypes), the majority of β* showed substantial similarity to their original compotypes, with more than 60% of cases showing H>0.8 (Fig 5). The overall high degree of similarity achieved across all three community detection algorithms indicates that the communities are able to successfully predict the composition of compotypes, while the eigenvector of β may represent something else (see S1 File, section: On the eigenvector of the full-β). Thus, compotype species can be successfully predicted based only on the complex chemistry that is in a β. A test to ascertain whether a better community-to-compotype assignment and prediction could be achieved at random was performed. The test measured (for each community-to-compotype assignment) the probability of achieving higher H values by a random community–a community with the same size as the assigned community but with different molecule types. The test was repeated 10^3^ times for each assigned community. The test showed that it is highly unlikely to achieve better H values by random community assignment (Table 1, and Fig E in S1 File).

**Table 1:** Statistics related to communities and compotypes.

		Louvain	Infomap	OSLOM	β
NC = 1	Mean	0.829±0.157	0.839±0.149	0.845±0.158	0.975±0.540
NC>1	Mean	0.797±0.217	0.823±0.171	0.747±0.254	0.624±0.269
Overall	Mean	0.805±0.204	0.827±0.166	0.773±0.237	0.716±0.280
Probability of a better similarity at random		0.0257±.135	0.00388±0.0156	0.0137±0.0399	
Size of assigned community		9±14	6±3	10±5	

Mean, standard deviation and percentage of dataset achieving high similarity between the eigenvectors of β* and full-β and the original compotypes (NC = 1, cases when single compotype observed; NC>1, cases when multiple compotypes observed; Overall, the entire dataset), for the three algorithms (Fig D in S1 File).

**Fig 6:**
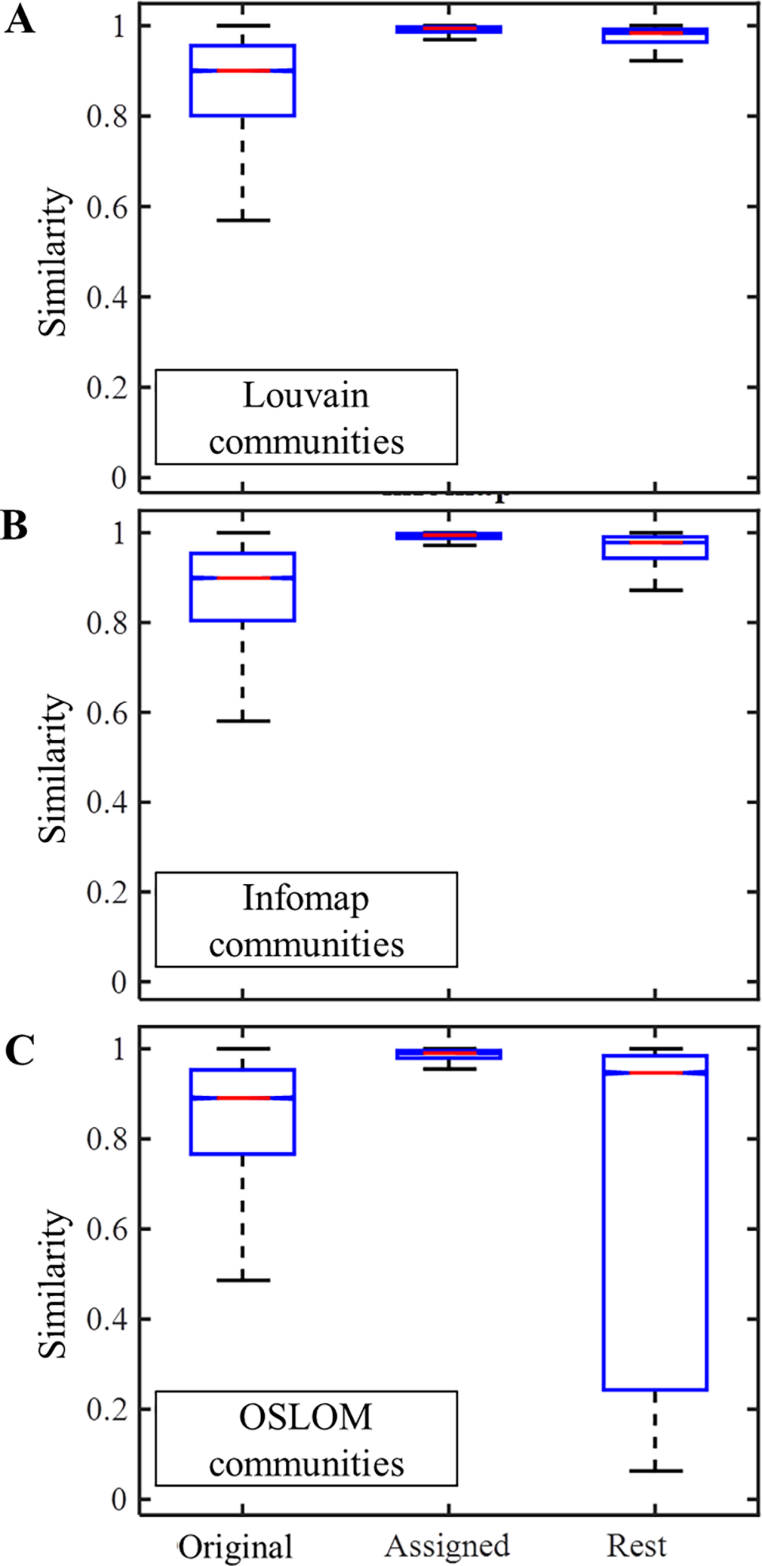
Box plots of compositional similarity, for the three community detection algorithms (Louvain, top; Infomap, middle; OSLOM, bottom). Similarity was measured in three cases: ‘*Original*’, when comparing the original compotype observed vs. the one in GARD under β* of its assigned community; ‘*Assigned*’, when comparing the compotype observed in a GARD simulation with β* of its assigned community to the eigenvector of β*; ‘*Rest*’, analogue to ‘Assigned’, only with communities that were not assigned to original compotypes β*. Mean and standard deviations for ‘*Original*’ respectively are: 0.849±0.165, 0.856±0.145 and 0.813±0.216.

Finally, it is important to verify whether indeed β* represents a meaningful chemistry that can give rise to a compotype species under GARD’s stochastic dynamics (Eq 1). To this end, GARD simulations were repeated with exactly the same parameters (see section: GARD simulations), and with β* for each assigned community rather than with the full β. Compotype identification in the new simulations was performed exactly as before (i.e., k-means clustering) and the compositional similarity to the original compotype was calculated (Fig 6 ‘Original’). A high similarity to the original compotype was always obtained, corroborating the community detection algorithms ability to detect the communities which serve as the ‘invariant content’ of GARD’s compotypes. In [81], the authors analysed stochastic Kauffman-like dynamics via the introduction of a temporal-window in order to determine which part of their reaction network is currently active, however, the novelty of the present paper is in enabling to make such determination *a-priori* based on the network topology.

As presently it is impossible to determine *a priori* the number of compotype species that will be observed, the algorithm for compotype-community assignment presented above is required to address all compotypes (however, it was previously shown that having an excess of mutual-interactions over self-interactions in β (i.e. β_ij_ over β_ii_) is a necessary but insufficient condition for a high number of compotypes [40]).

### On the nature of non-assigned communities

**Fig 7:**
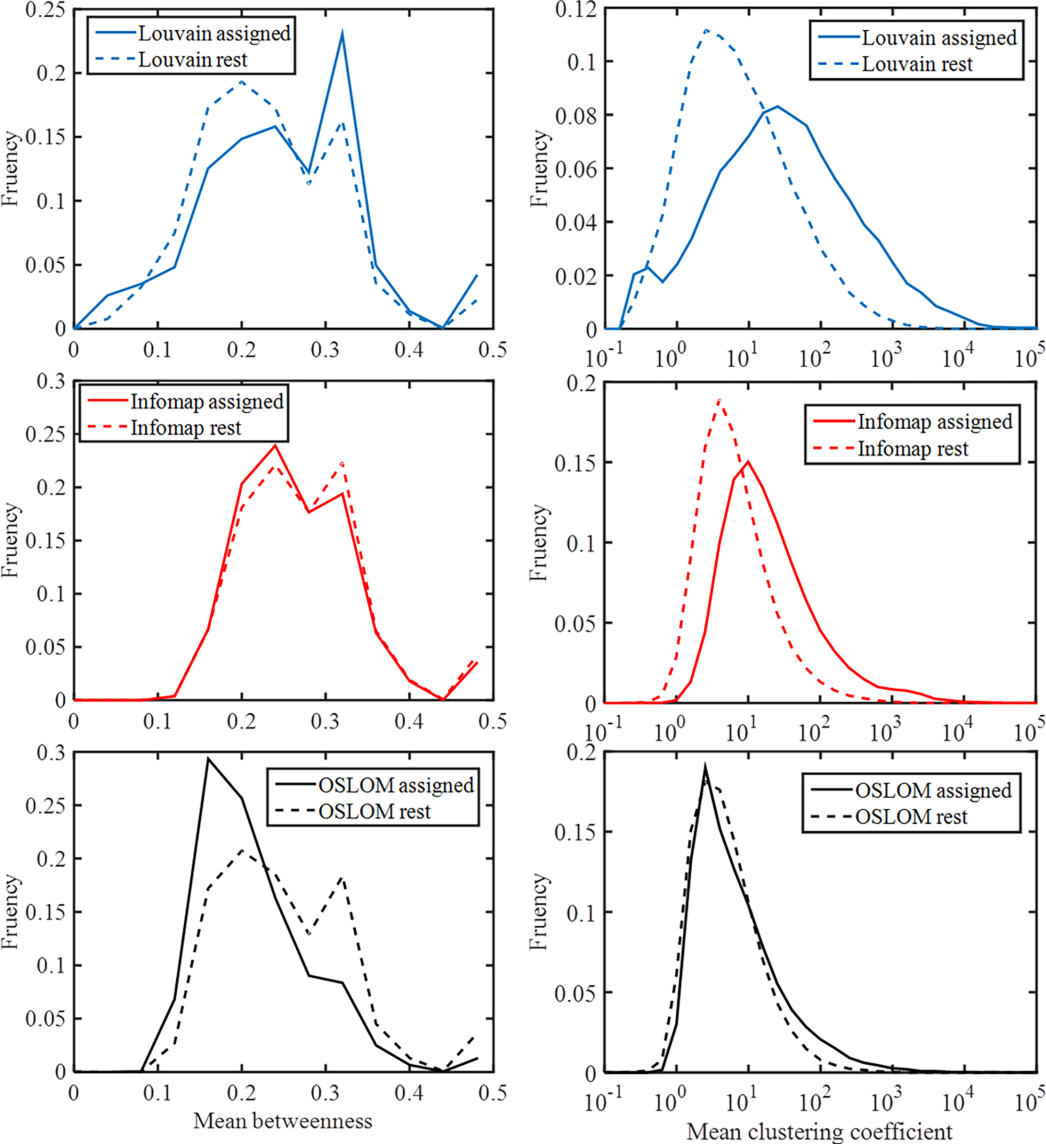
Network topology for assigned communities and rest, for the three community-detection algorithms (Louvain, top; Infomap, middle, OSLOM, bottom). (left) Node-betweenness-centrality [82], normalized by dividing with (n-1)*(n-2), where n is number of nodes in a community. (right) Clustering-coefficient [83]. Parameters were calculated using [84].

Lastly, it is asked why some communities successfully predict compotypes while other communities do not, and are there differences between those communities. It was previously suggested that compotype dynamics are somehow related to the compartments formed by high β_ij_ values [44]. The morphology of the communities assigned to compotypes seems to be different than that of those which were not assigned (Fig 7), which may begin to point to the nature of differences between the assigned and non-assigned detected communities. Additionally, the similarity between the eigenvector of β* and the compotype from GARD under β* was calculated, both for the assigned and non-assigned communities. It was found that this similarity is much higher for the assigned communities than for the non-assigned (Fig 6 ‘Assigned’ and ‘Rest’). This last result suggests that the dynamics of the non-assigned communities is fundamentally different than that of the assigned ones, in the sense that the former are less likely to exhibit faithful replication. An ongoing investigation is on its way to further understand those differences, which may prove critical for reverse-engineering, i.e. the design of a β network that give rise to specific and desired compotypes dynamics.

## Conclusions

The GARD model performs biased and far from equilibrium random walks on a network that has previously been linked to pre-biotic evolutionary dynamics. Via community analyses, we were able to bypass the dynamic trajectories of the stochastic simulator and use the ensemble of detected communities to predict the emergence of (proto) species of this system as well as their invariant content. Interestingly, the morphology of assigned communities is different than that of non-assigned ones, which deserve further scrutiny in order to understand the nature of this difference, how the various topological characteristics affect dynamics as well as the precise role of those un-assigned communities.

We have used the eigenvector of β* to predict compotypes and corroborated by performing GARD dynamics under β*, to find that GARD-dynamics approach gives rise to a compotype more similar to the original one (the original compotype observed under the full-β). In other words: using β*, GARD-dynamics are ‘closer to the truth’. This is both non-intuitive and interesting, because the eigenvector approach does not employ GARD’s stochastic dynamics, where the latter are expected to introduce some variation in the compotype content. If we treat the observation of species in GARD’s dynamics as the ground truth–analogous to how species are observed in nature–then this points that the theoretical prediction using the eigenvector is imperfect (but still very good!), probably because the eigenvector method takes into account only β and not the full physio-chemical details of the GARD model, such as the reversibility of assembly-joining.

For tractability, the present manuscript kept to the definition and identification of compotype species as they have traditionally been used in GARD and lipid world literature [32, 40–42, 80]. We would like to argue in favour of rethinking species identification, as follows. We speculate that the un-assigned communities represent either assemblies that are unable to faithfully replicate or compotype species that are very rare. The latter may require an even larger scale simulation analysis than the one we have done here involving more runs and longer simulation times before these rare species could be observed. Any species identification algorithm developed must, critically, acknowledge faithful replication. As presently it is impossible to determine a-priori the number of compotype species that will be observed in a simulation under a given β network, we are in the process of extending this current paper in order to precisely predict the expected number of compotype species under a given β without running simulations. The community count provides an upper limit for the species count, and the community eigenvectors, even if somewhat numerous, still strongly narrows the search for compotypes.

Our heuristic approach gave very similar results among all three community-detection-algorithms we used, thus providing robustness to our findings. Future extension of this work will apply the species-prediction-algorithm developed herein on multiple dynamical models and their emergent species (or species equivalent), as well as address larger networks which is more realistic, in order to address the generality of the algorithm presented here.

## Supporting information

S1 File Supplementary data and figures associated with this article. (PDF)
