# Supplementary material for: Predicting species emergence in simulated complex pre-biotic networks
Source: PLoS One. 2018 Feb 15;13(2):e0192871. doi: 10.1371/journal.pone.0192871 (PMC5813963; doi:10.1371/journal.pone.0192871)
Supplement: S1 File — (PDF) [file pone.0192871.s001.pdf]

Predicting species emergence in simulated complex pre-biotic networks: Supporting information

Omer Markovitch and Natalio Krasnogor*
omermar@gmail.com ; natalio.krasnogor@newcastle.ac.uk

1. MATLAB code

GARD simulations were ran using GARD10 MATLAB code [Markovitch, O. and Lancet, D. Excess Mutual Catalysis Is Required for Effective Evolvability. Artificial Life 18, 3 (2012) (DOI: 10.1162/artl_a_00064)]. The following code is provided as attachment to the present manuscript:

Generating a lognormal β and performing community detection:

- Beta_louvain.m; receives a random-seed and generates a lognormal β using GARD10, then Louvain's MATLAB function *cluster_jl_orient* is run. Output out.indices is an array holding for each molecule the index of the community it belongs to.
- Beta_infomap.m: receives a random-seed and generates a lognormal β using GARD10, then saves the β to disk and run *Infomap* as an externally program (UNIX or Windows). Output out.indices is an array holding for each molecule the index of the community it belongs to.
- Beta_oslom.m: similar to Beta_infomap.m, running program *oslom_dir*. Ouput out.indices is an array of *cells*, holding for each molecule a list of indices of communities it belongs to.

getmaxrealevec.m receives a β matrix (or β*) and return as its first output the eigenvector with the highest real eigenvalue.

Are all available at http://ico2s.org/data/extras/gard/ and [Markovitch, O. and Krasnogor, N. Accompanying dataset for: Predicting Species Emergence in Simulated Complex Pre-Biotic Networks. Zenodo (2016) (DOI: 10.5281/zenodo.56534)]

2. Accompanying dataset

Full dataset with the entire 10,000 networks and simulations analysed is available freely at http://ico2s.org/data/extras/gard/ and [Markovitch, O. and Krasnogor, N. Accompanying dataset for: Predicting Species Emergence in Simulated Complex Pre-Biotic Networks. Zenodo (2016) (DOI: 10.5281/zenodo.56534)]. The dataset includes:

2.1. Images such as Fig 3 in the main text, for 100 cases studied for GARD tapes.

2.2. For each of the 10,000 β's and simulations used in the rest of the paper a text file with the following details:

2.2.1. Seed number that was used to generate the specific β network

2.2.2. For each compotype observed in the GARD simulation under this β – a vector of its composition (elements normalized to have sum = 1.0).

2.3. For each of the 3 community detection algorithms (Louvain, Infomap and OSLOM):

2.3.1. Indices of which communities were assigned to which compotype.

2.3.2. For each detected community – a list of the indices stating for each node to which community it belongs to.

2.4. A Matrix with full values of β.

3. GARD tapes

For each β, 9 additional simulation-runs (tapes) were repeated, and in each tape the compotypes were identified as before (i.e. k-means clustering). Fig A shows a histogram of the number of compotype species observed under each β of the 100 studied for this part, vs. the first tape. The similarity (H, Eq 3 in the main text) between the compotypes identified in each simulation-run to the first simulation-run was also calculated (Fig B).

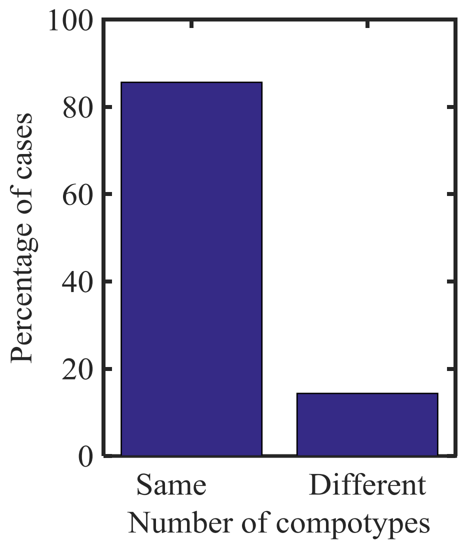

Fig A. Compotype count with respect to the first tape. Bar plot of the number of cases a tape showed same or different number of compotype species than the first tape, for the 100 β's studied for this part. %(same)=85.66.

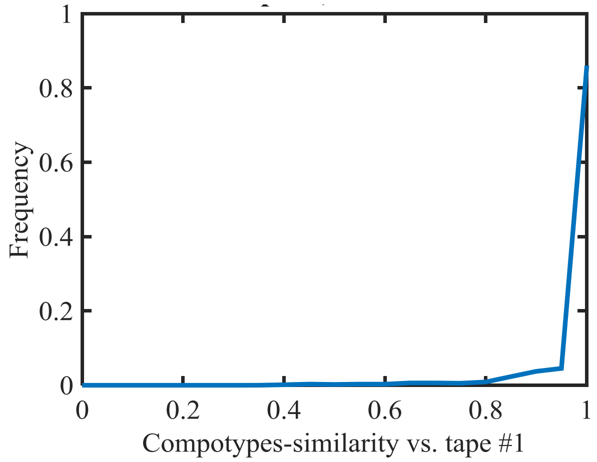

Fig B. Compotypes similarity (H, Eq 3 in the main text) with respect to the first tape. Histogram of similarity between compotypes identified at different tapes to those identified in tape #1. Mean = 0.976±0.0747.

4. Histograms of angle to the eigenvector of the full-β and β*

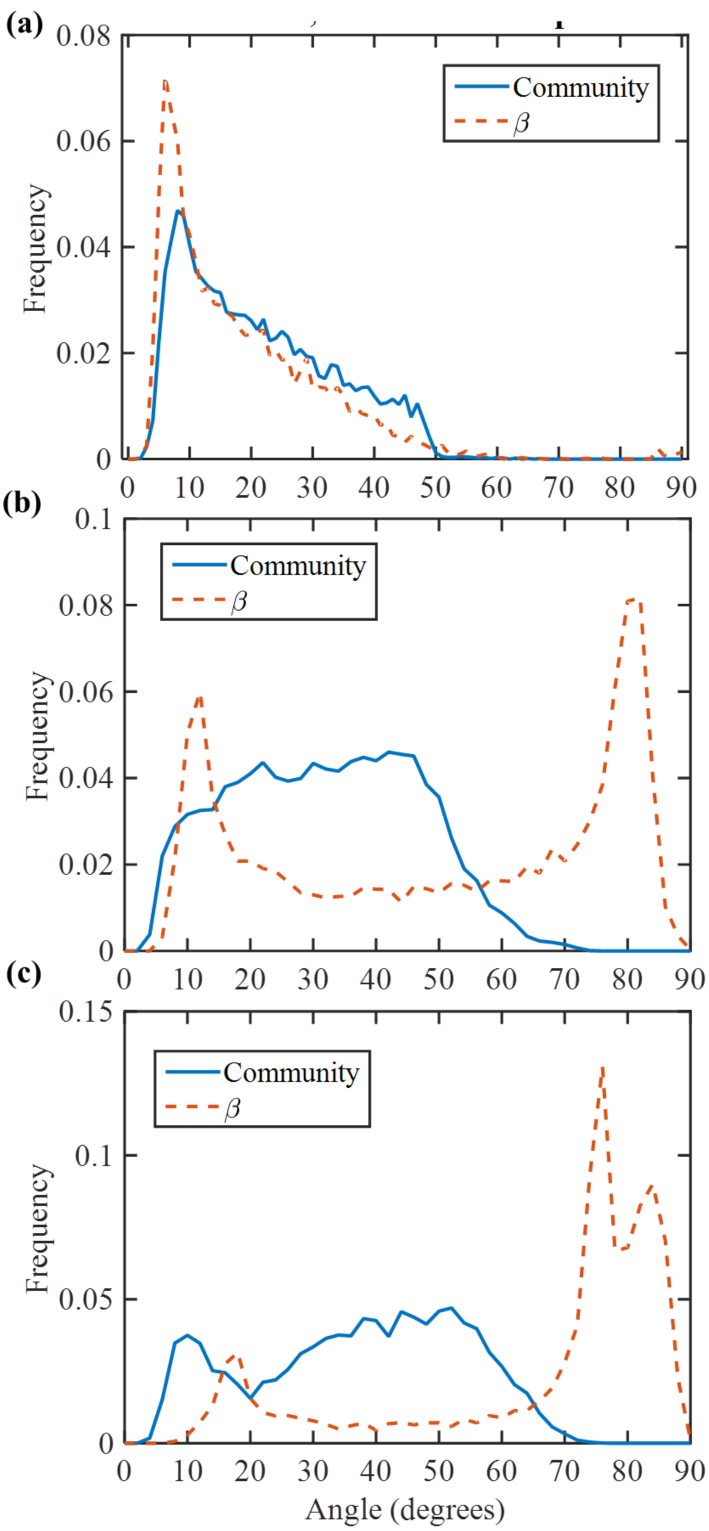

Fig C. Angles between assemblies and eigenvectors. Histograms of the angles between the assemblies and the eigenvector of β***** (Eq 2 in the main text) and the full β, for the three examples given in Fig 3 in the main text. Means and standard deviations are given in Table S1.

Panel	Community	β
(a)	21±12	18±13
(b)	32±15	51±27
(c)	37±17	66±22

Table S1. Mean and standard deviations of angle distributions in Fig C.

5. Compotype-community assignment

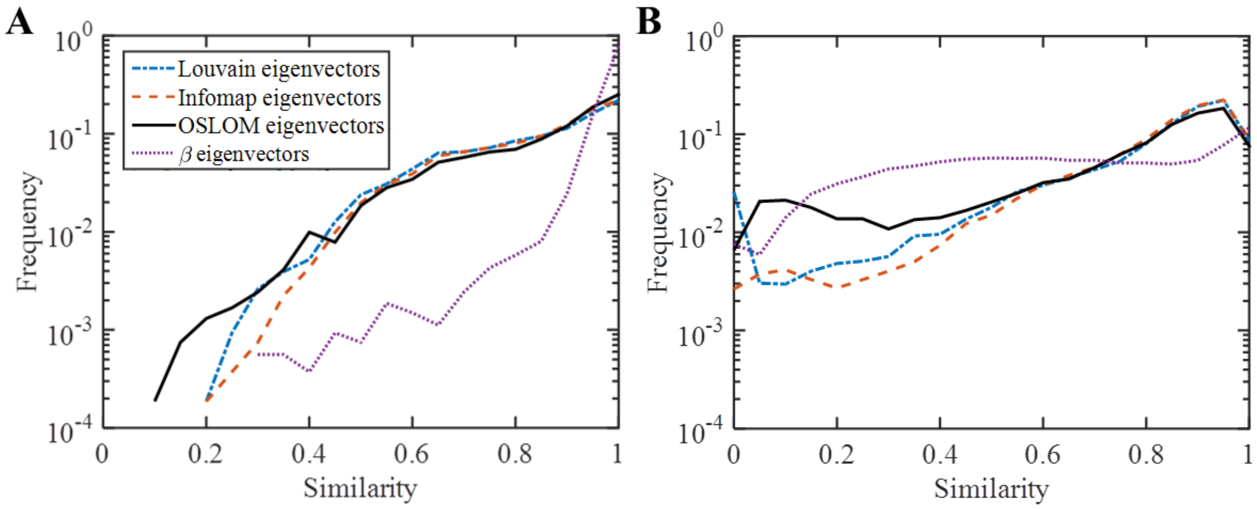

Fig D. Histograms of compositional similarity when predicting compotypes using the eigenvectors of β* and full-β. (A) Comparing all the cases when a single compotype was observed (NC=1). (B) Comparing all the compotypes from all the cases were two or more compotypes were observed (NC>1). Mean and standard deviation are given in Table 1 in the main text.

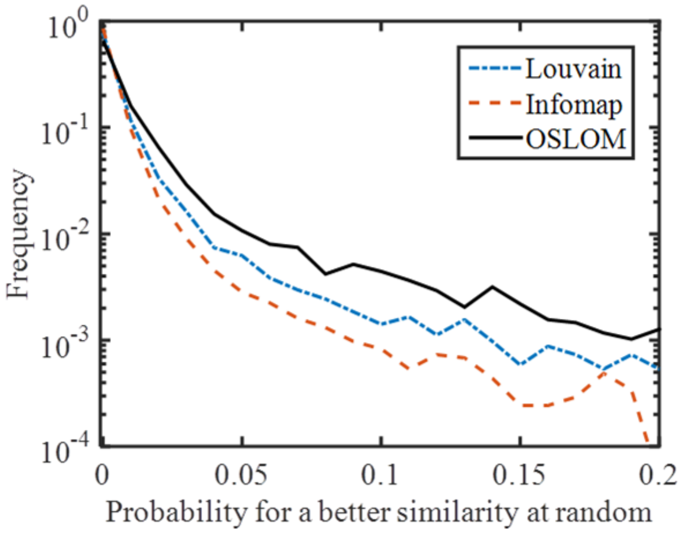

Fig E. Testing for random assignment of compotype-community. Histogram of the probability of achieving a higher similarity by a random community (figure truncated at p=0.2). Mean and standard deviations are given in Table 1 in the main text.

6. On the eigenvector of the full-β

In the limiting case, where only a single compotype was observed, using Perron-Frobenius theorem directly on the full-β resulted in an eigenvector which was highly similar to that compotype (Fig F). When multiple compotypes were observed in a simulation under a given β, the average similarity between eigenvector of the full-β to each of those compotypes was low, yet the similarity to the average-compotype was quite high (Fig F). The average-compotype is a vector whose elements are the component-wise average of the compotypes that emerged under a given β. It follows that the eigenvector of the full-β is the center of mass of the entire assemblies in the simulation, and it is suggested that the eigenvectors of the different β* represent potential attractors in the compositional space (i.e. the space of all possible compositions of N_{max} molecules out of an alphabet of N_G molecular types). As a control, the similarity between the eigenvectors of β* and the average-β*-eigenvectors to the average-compotype were calculated, as well as between the average-β*-eigenvectors to the eigenvector of the full-β and all found to be substantially low (Fig G). The average-β*-eigenvectors is a vector whose elements are the component-wise average of all the eigenvectors of all the communities detected under a given β.

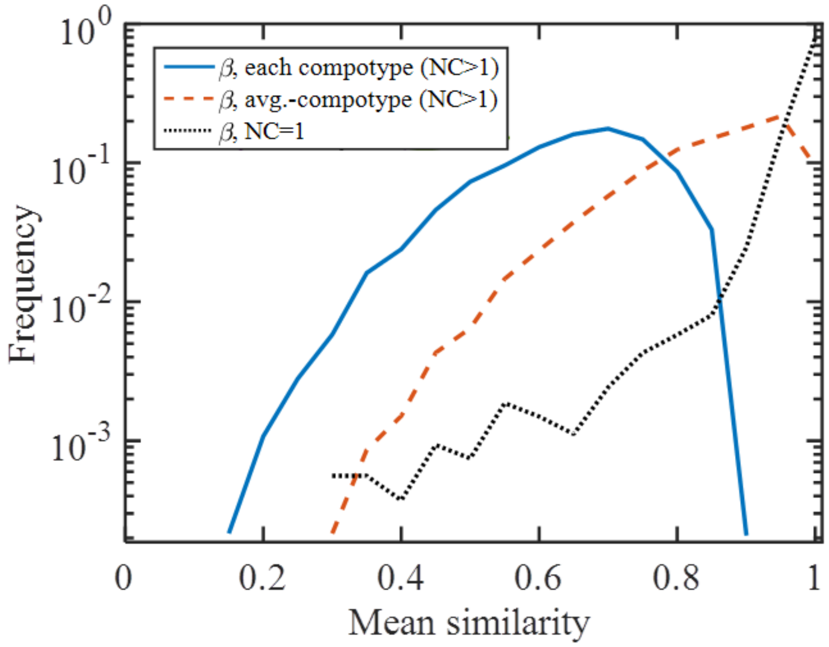

Fig F. Histograms of similarity with respect to the eigenvector of the full-β. The average of similarity to each of the compotypes when multiple compotypes observed (blue solid line), the similarity to the average-compotype when multiple compotypes observed (red broken line) and when only a single compotype is observed (black dotted line). Mean values respectively are: 0.642±0.119, 0.848±0.116 and 0.975±0.054.

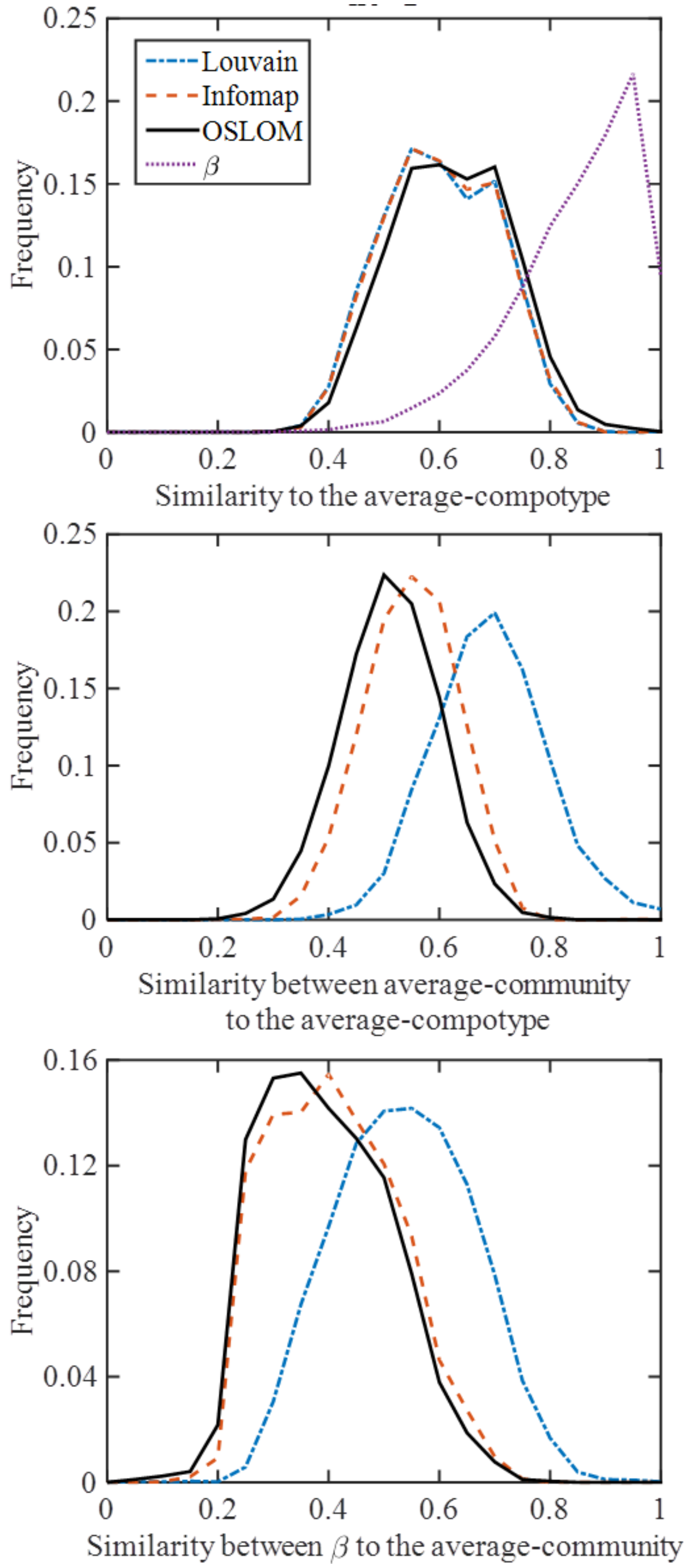

Fig G. Similarities with respect to the average-compotype. (top) Similarity between the eigenvector of β* (the one that gives the highest similarity is picked) to the average-compotype. For reference the similarity with the eigenvector of the full-β is also shown (Fig F). (middle) Similarity between the average-β*-eigenvector to the average-compotype. (bottom) Similarity between the eigenvector of the full-β to the average-β*-eigenvector.
